# Effect of Self-Esteem and Parents’ Psychological Control on the Relationship Between Teacher Support and Chinese Migrant Children’s Academic Achievement: A Moderated Mediation

**DOI:** 10.3389/fpsyg.2019.02342

**Published:** 2019-11-01

**Authors:** Guirong Liu, Xiuqin Teng, Dongchun Zhu

**Affiliations:** ^1^Department of Teacher Education, Qilu Normal University, Jinan, China; ^2^Shandong Dongheng Colloidal Material Co., Ltd., Jinan, China

**Keywords:** teacher support, academic achievement, positive self-esteem, self-deprecation, parents’ psychological control

## Abstract

Based on Self-Determination Theory (SDT) and Ecological Systems Theory (EST), this study attempted to examine the relationship between teacher support and Chinese migrant children’s academic achievement as well as the mediating role of self-esteem and the moderating role of parents’ psychological control. An opt-in consent procedure was employed and participation rates were 85%. Finally, six hundred and one migrant children participated in the study in spring and completed self-report questionnaires concerning teacher support, self-esteem and parents’ psychological control. Teacher support was measured by Teacher Behavior Questionnaire, self-esteem by Rosenberg’s Self-esteem Scale, and parents’ psychological control by 18 items from prior research. And students’ final academic performance of the semester was provided by the dean office. Results indicated that self-esteem (positive self-esteem/self-deprecation) mediated the positive relationship between teacher support and academic achievement. Parents’ psychological control moderated the relationship between teacher support and self-deprecation. Furthermore, parents’ psychological control moderated the mediating effect of self-deprecation on teacher support–academic achievement relationship, such that the mediating effect was positive when the level of psychological control was high while the mediating effect was not significant when psychological control was low. Parents’ psychological control neither moderate teacher support – positive self-esteem link nor the mediating effect. The findings are consistent with SDT and EST, and have both culturally specific and universal meanings. The implications of the study for promoting Chinese migrant children’s academic achievement are also discussed.

## Introduction

With the rapid development of urbanization, China’s large-scale and persistent rural-to-urban migration has taken place in the past three decades. By the end of 2015, more than 253 million migrant workers have moved from rural to urban areas in search of better living conditions (Population Census Office under the State Council, 2015). With the increasing prominence of family migration, the population of children in compulsory education stage was 12.95 million (Ministry of Education of the People’s Republic of China, 2015) and the number is still rising. The migrating and moving had great influence on migrant children’s original lives, which brought great challenges to their identity development (Salmeri and Pellerone, 2015) and academic function. Considered as family’s face and a short cut to upper social stratum, academic achievement is paid high attention by parents, just as the proverb says, “***wan ban jie xia pin, wei you du shu gao***”(that is, to be a scholar is to be the top of society). Thus education was taken as top priority when they decided to migrate (Shen et al., 2015; Wong et al., 2015). The current research attempted to explore the contextual and individual determinants of migrant children’s academic achievement.

### The Role of Teacher Support in Academic Achievement

Due to living and working pressure, the parents of migrant children provide children little support (Zeng, 2010). Teachers have the potential to be an important source of support (Metheny et al., 2008), for children spend large amount of time learning and living at school. Empirical studies have repeatedly confirmed that teacher support was positively associated with academic achievement (Malecki and Demaray, 2003; Sakiz et al., 2012; Ruzek et al., 2016; Strati et al., 2016; Liu and Zhang, 2017). Moreover, teachers have greater effect on the students most in need (Mercer et al., 2011), so are the migrant children, a vulnerable population (Wu et al., 2010). For this marginalized group, a teacher is “just one person” (Igoa, 1995), who makes a positive impact on migrant children’s lives (Borjian and Padilla, 2010). The primary purpose of the study was to analyze the potential “path” from teacher support to migrant children’s academic achievement.

### The Mediating Effect of Self-Esteem

Based on Self-Determination Theory (SDT), there are three human basic needs: competence, autonomy, and relatedness. Self-esteem is an integrity understanding of an individual’s capacity and self-value (Pyszczynski et al., 2004). SDT emphasizes the importance of environments in children’s internal motivation. In particular, a supportive environment (e.g., teacher support) can enhance intrinsic motivation, curiosity, capacity and longing for challenge (Ryan, 1982; Flink et al., 1990). Compared to non-immigrant adolescents, care from mothers was much more important for immigrants’ self-esteem (Miconi et al., 2017). For language brokering Mexican American emerging adults, support was positively related to self-esteem while conflict and parental alienation were negatively associated with self-esteem (Weisskirch, 2013). Perceived discrimination negatively predicted South American immigrants’ self-esteem (Urzua et al., 2018).

Self-esteem positively predicted academic achievement of diverse samples, such as university students, pre-university students, adolescents, and so on (Roman et al., 2008; Aryana, 2010; Li et al., 2018; Saha and Tamanna, 2018). Self-esteem is particularly conducive to educational achievement among minority youths or immigrant adolescents (Carranza et al., 2009). Li et al. (2018) found that self-esteem fully mediated the relationship between social support and university students’ academic achievement. Then it is expected that self-esteem also mediates the positive relationship between teacher support and Chinese migrant children’s academic achievement.

### The Moderating Effect of Parents’ Psychological Control

In comparison with schooling and other environmental factors, parents, an important contextual factor, act as the stable and sustaining source (Steinberg et al., 1992; Liu et al., 2013). The impact of parenting on immigrant/migrant children has been accepted universally (Miconi et al., 2017; Xu et al., 2017; Carlo et al., 2018). Parents’ psychological control is a manipulative and suppressing parenting, including authority assertion, love withdrawal, and guilt induction on children’s psychological world (Barber et al., 2005; Sternberg, 2005). The detrimental impact of psychological control is that it violates children’s self-sense. Specifically, psychological control chiefly attenuates children’s emotional functioning and consequently leads to undesirable outcomes, such as externalizing problems (Barber and Harmon, 2002) and internal problems, such as loneliness, depression, emotional distress, and diminished self-esteem (Barber, 1996; Silk et al., 2003; Wang et al., 2007; Chang, 2011). Studies of parenting in Chinese migrant families found that, parents tended to adopt negative parenting styles such as punishment and authoritarian, and seldom treated children with positive parenting styles such as emotional warmth and understanding (Ma et al., 2015).

We argue that psychological control is a particularly important construct to examine as a moderator for two reasons. First, Bronfenbrenner’s classical Ecological Systems Theory (EST) suggests that, to understand person-context interrelatedness, it is necessary to examine the relations among microsystem, mesosystem, exosystem, macrosystem, and chronosystem frameworks (Bronfenbrenner, 1977). Specifically, EST includes not only person (e.g., person’s self-esteem, self-efficacy, self-confidence), contexts (school, family), processes of interaction (person-context interaction) and time, but also the interactions of different subsystems (Tudge et al., 2009). As underscored in the EST, the interactions of person-context and different subsystems were significant for an individual’s development, so the elements in the same system may interact with each other. In a similar way, teacher support and parental psychological control might interact with each other on self-esteem. In other words, the relationship between teacher support and self-esteem depends on the level of psychological control.

Second, while esteem can be nurtured by supporting environments, such as maternal care, social support and so on (Miconi et al., 2017; Li et al., 2018), it was vulnerable to disadvantaged contexts, such as parental alienation, conflict, perceived discrimination, and so on (Weisskirch, 2013; Urzua et al., 2018). And Sun et al. (2019) found that parental psychological control negatively predicts adolescents’ self-esteem. So, it is plausible to suggest that the relation between teacher support and self-esteem may depend on the level of psychological control.

### The Moderated Mediation

The prior arguments represent an integrated framework in which self-esteem mediates the positive relation between teacher support and academic achievement and psychological control moderates the relation between teacher support and self-esteem. According to the notion that psychological control moderates the relation between teacher support and self-esteem, and considering that self-esteem is related to academic achievement, it is logical to propose that psychological control also moderates the mediating mechanism for self-esteem in the relation between teacher support and academic achievement – a mediated moderation model (Edwards and Lambert, 2007).

To sum up, based on SDT and EST, we attempted to investigate the simultaneous influence of contextual factors (teacher support, psychological control) and individual factor (self-esteem), which matter for migrant children’s academic achievement.

However, the issues such as the following need to be addressed.

Firstly, most research took self-esteem as a continuum, and found that self-esteem played as a positive factor of academic achievement (Roman et al., 2008; Aryana, 2010; Li et al., 2018; Saha and Tamanna, 2018). There were exceptions, however. For example, Vialle et al. (2015) found no correlation between self-esteem and academic achievement for the gifted group. Lew and Harklau (2018) indicated high self-esteem had a negative effect on children’s academic achievement. These results were inconsistent with previous research that high self-esteem were positively related to children’s academic achievement (Saha and Tamanna, 2018). This result might be related to one dimensional conceptualization of self-esteem. To date, few research is conducted from two or multiple dimensions of self-esteem, and the classification of self-esteem as positive self-esteem and self-deprecation is such dichotomous conceptualization (Frank et al., 2010; Danniel et al., 2016). An individual could own feelings of positive self-esteem and self-deprecation simultaneously (Owens, 1994; Boucher et al., 2009). Positive self-esteem is an active and steady sense of self-worthiness and competence, whereas self-deprecation means inferior or inadequate (Rosenberg, 1979). Frank et al. (2010) proved the differential impact of positive self-esteem and self-deprecation on general self-efficacy. Value-differentiation was negatively associated with positive self-esteem, especially for immigrant youth (Danniel et al., 2016). Bakhtiar et al. (2017) found that parent-child conflict was positively related with to self-deprecation for Zoroastrian young adults in immigrant families while meeting parental expectations predicted self-deprecation negatively. Furthermore, Teng et al. (2017) found positive self-esteem and self-deprecation partially mediated the relationship between teacher support and Chinese migrant children’s academic achievement, respectively. In our current research, self-esteem is conceptualized in terms of two dimensions: positive self-esteem and self-deprecation. And teacher support was supposed to positively predict positive self-esteem, and in turn, improve academic achievement; teacher support was hypothesized to be negatively related to self-deprecation, and then, negatively predicted academic achievement.

Secondly, to our knowledge, prior research examined the significance of teachers (Metheny et al., 2008; Sakiz et al., 2012; Ruzek et al., 2016; Strati et al., 2016) or parents, respectively (Carranza et al., 2009; Frank et al., 2010; Cheung and Pomerantz, 2011; Miconi et al., 2017) or their parallel function (Wu et al., 2010; Hampden-Thompson and Galindo, 2017). However, the research on interaction of both parents and teachers is sparse. EST offers us a new perspective, but empirical research is rare. Hence, we attempted to explore the simultaneous influence of teachers and parents on academic achievement.

Finally, in current research on parental control and autonomy support practices, there is debate and controversy about whether associations between psychological control and ill-being generalize across cultures (Soenens and Beyers, 2012). On the one hand, although previous studies in the west have shown the negative relationship among psychological control and academic achievement (Jeon, 2007; Chang, 2011; Lee et al., 2016), psychological control are more commonly endorsed by Chinese parents and migrant families in particular. Because those practices are widespread or even normative, they would be less detrimental. What’s more, in China, Confucian culture is the most influential culture, which emphasizes collectivism and interdependence. Within the framework of dominant Confucian culture, children generally construe the notion “Guan” as “to love,” “to govern,” and “to train” (Chao, 1994; Wang et al., 2007). The detrimental impact of psychological control may be attenuated by interpreting parents’ intention as “doing good for the child” or “being right” (Helweig et al., 2014).

On the other hand, studies increasingly show that parental psychological control is related to ill-being and adverse developmental outcomes across cultures (Barber et al., 2005) while autonomy is also the basic need of Collectivistically Oriented Korean students (Jang et al., 2009).

On the whole, there are competing propositions regarding the role of psychological control. Considering the vulnerable context migrant children grow up, we are prone to agree with the first, culture-specific hypotheses.

### Hypotheses and Conceptual Framework

In sum, the current study is aimed at addressing four major research questions:

(1) How do positive self-esteem and self-deprecation mediate the effect of teacher support on academic achievement, respectively?
(2) How does psychological control moderate the effect of teacher support on positive self-esteem and self-deprecation, respectively?
(3) How does psychological control moderate the mediating effect of positive self-esteem and self-deprecation on teacher support–academic achievement relationship, respectively?

Correspondingly, the hypotheses of the article are as following:

Hypothesis 1: Self-esteem mediated the positive relation between teacher support and academic achievement. To be specific, teacher support was positively related to positive self-esteem, and then, predict better academic achievement. While teacher support was negatively related to self-deprecation, and then, predicted lower academic achievement.
Hypothesis 2: Parents psychological control moderated the relation between teacher support and self-esteem.
Hyperthesis3: Parents psychological control moderated the mediating effect of self-esteem on the teacher support – academic achievement relationship.

**FIGURE 1:**
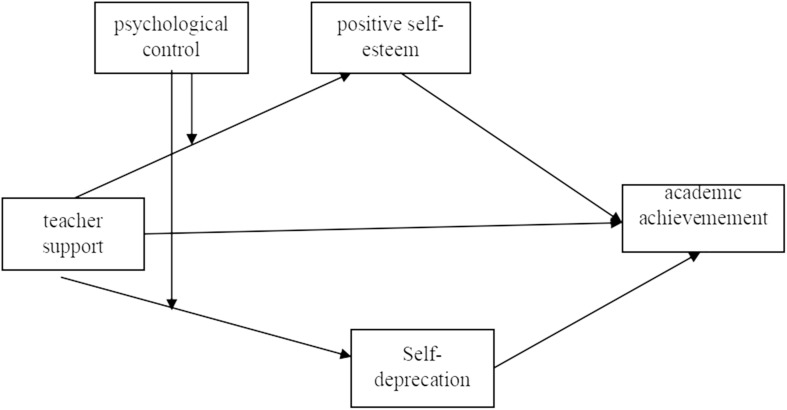
Conceptual model.

The present research develops a conceptual framework linking teacher support and academic achievement. The paper develops propositions about the mediating effect of self-esteem, the moderating effect of psychological control, and the overall moderated mediation (Figure 1).

## Materials and Methods

### Participants

The participants in this study were 601 pupils in Jinan, China. The pupils were attending two migrant primary schools that served the fourth, fifth, and sixth graders in two public school districts, one of which (179) near a major metropolitan area is above average achievement level, the other (422) is in rural-urban fringe zone of average. Of the 601 participants, 307 (51.1%) were boys and 294 (48.9%) were girls; 221 (36.8%) were fourth graders, 206 (34.3%) were fifth graders and 174 (28.9%) were six graders. Participants’ mean age was 11.30 years (*SD* = 0.86). In terms of income, parents’ median income ranged from ¥8000 to ¥10,000 per month. In the aspect of education, about 25% of the parents completed high school or less, 70% had a college/university education, and 5% finished postgraduate education. Concerning occupation, 75% of the parents were self-employed, and 15% were employed in factories, and 10% held a professional, managerial or technical position.

### Procedure

The design of this study was reviewed and approved by the Institutional Review Board at Qilu Normal University School approval. In the two migrant elementary schools, those children whose domicile place was in Jinan were excluded from our survey. With regard to those qualified children, before collecting questionnaires, parental written informed consents for their children to participate were obtained. And finally, 85% of the parents permitted their children’s participation in our research. Surveys lasted about 40 min. Two research assistants in one classroom administered the survey, monitored the progress of the participants, and gave instructions if the participants had anything unclear. Participants were assured that the information they provided would be absolutely confidential. Research assistants asked participants to provide student numbers and grade for crediting purposes.

After surveys were completed, participants received a token gift, of small monetary value.

### Measures

#### Teacher Support

Teacher Behavior Questionnaire developed by Ouyang and Song (2005) was adopted to assess the extent to which students perceive their teachers’ support. The questionnaire contains 15 items including three subscales: (a) perceived teacher learning support (4 items, e.g., when I give wrong answers, the teacher will often explain the reason why I did wrong and tell me how to correct them); (b) perceived teacher affective support (5 items, e.g., the teacher asks me to answer questions with encouraging eyes); and (c) perceived teacher competence support (6 items, e.g., the teacher believes me that I can always complete difficult task or assignment). Correlational research showed the inventory has a good reliability and validity among Chinese children (Qiao et al., 2013). In the present research, the questionnaire demonstrated a good internal consistency, with Cronbach’s alpha coefficients of 0.71, 0.79, and 0.85, respectively. Confirmatory factor analysis (CFA) has shown an excellent fit of the model: χ*^2^/df* = 3.48, RMSEA = 0.06, TLI = 0.97, CFI = 0.98.

#### Self-Esteem

Rosenberg’s Self-esteem Scale (Rosenberg, 1965) was used to assess students’ self-esteem. The questionnaire consists of 10 items, 6 items assessing positive self-esteem, e.g., in general, I am satisfactory with myself; 4 items assessing self-deprecation, e.g., in a word, I am inclined to take myself as a failure. The items are rated on a 4-point Likert scale from 1 (not at all true) to 4 (very true). CFA of the scale has shown a good fit of the model: χ*^2^/df* = 2.87, RMSEA = 0.06, TLI = 0.96, CFI = 0.97.

#### Parents’ Psychological Control

Parents’ psychological control was measured by 18 items selected from prior research (Silk et al., 2003; Wang et al., 2007; Cheung and Pomerantz, 2011). Using 5-point Likert Scale from 1 (not at all true) to 5 (very true), children report the extent to which they perceive parents’ psychological control, which are divided by three subscales, (a) guilt induction (10 items, e.g., my parents tell me that I should feel ashamed when I do not behave as they wish); (b) love withdrawal (5 items, e.g., my parents avoid looking at me when I have disappointed them); and (c) authority assertion (e.g., 3 items, my parents say when I grow up, I will surely appreciate all the decisions they have made for me). Mean scores of the 18 items are calculated, higher scores mean parents’ higher psychological control. CFA of the scale has shown an adequate fit of the model: χ*^2^*/*df* = 4.20, RMSEA = 0.07, TLI = 0.92, CFI = 0.93.

#### Academic Achievement

Following studies about academic achievement (Chen et al., 2010; Yang et al., 2019), students’ scores for Chinese, Math, and English on the final exams for the semester were obtained from schools and standardized within each class. Then, the standardized scores were averaged to represent students’ academic achievement.

## Results

### Descriptive Statistics and Correlations

Table 1 presents the means, standard deviations, and correlations of all key variables. As was shown in Table 1, teacher support was positively correlated with positive self-esteem (*r* = 0.44, *p* < 0.001) and academic achievement (*r* = 0.30, *p* < 0.001). Moreover, positive self-esteem was positively correlated with academic achievement (*r* = 0.25, *p* < 0.001). These results provide initial support for our hypotheses.

**TABLE 1:** Means, standard deviations, and correlations among study variables.

**Variable**	***M***	***SD***	**1**	**2**	**3**	**4**	**5**	**6**	**7**
1. Gender^a^	0.51	0.50	–						
2. Age	11.30	0.86	0.03	–					
3. TS	3.76	0.82	−0.09^∗^	−0.09^∗^	(0.91)				
4. PC	2.88	0.84	0.18^∗∗∗^	0.01	−0.09^∗^	(0.84)			
5. PSE	3.03	0.56	0.03	−0.01	0.44^∗∗∗^	0.03	(0.73)		
6. SD	1.93	0.70	0.01	0.08	−0.24^∗∗∗^	0.41^∗∗∗^	−0.33^∗∗∗^	(0.76)	
7. AA	0	2.63	−0.16^∗∗∗^	−0.07	0.30^∗∗∗^	−0.18^∗∗∗^	0.25^∗∗∗^	−0.24^∗∗∗^	(0.84)

**N* = 601. Values in parentheses on the diagonal are the Cronbach’s alpha value of each scale. TS = teacher support; PC = psychological control; PSE = positive self-esteem; SD = self-deprecation; AA = academic achievement. ^*a*^Females were coded as 0 and males were coded as 1. ^∗^*p* < 0.05, two-tailed. ^∗∗^*p* < 0.01, two-tailed. ^∗∗∗^*p* < 0.001, two-tailed.*

### Testing Measurement Models

As presented in Table 1, the reliability of the measures showed a good internal consistency (>0.70). To assess the construct validity of the measures, a CFA was conducted with the proposed five factors. This model achieved good model fit (χ*^2^*/*df* = 2.50, RMSEA = 0.05, TLI = 0.94, CFI = 0.95).

### Hypothesis Testing

Path modeling was applied to test the hypothesis, due to its strength in testing complex models involving mediations and moderations. To account for the measurement errors for the latent measures, the factor scores were obtained from the proposed measurement model. The coefficients of the path model of positive self-esteem and self-deprecation were shown in Table 2.

**TABLE 2:** Path modeling estimation of coefficients.

**PSE as a mediator**		**SD as a mediator**	
**Direct relationship**			
TS → PSE	0.44^∗∗∗^	TS → SD	−0.20^∗∗∗^
PSE → AA	0.14^∗∗^	SD→ AA	−0.15^∗∗∗^
PC → PSE	–0.07	PC → SD	0.37^∗∗∗^
TS × PC → PSE	–0.03	TS × PC → SD	0.12^∗^
TS → AA	0.24^∗∗∗^	TS → AA	0.27^∗∗∗^
**Indirect path coefficients**			
TS → PSE → AA			0.06^∗∗^
TS → SD → AA			0.03^∗∗^

**N* = 601. TS = teacher support; PC = psychological control; PSE = positive self-esteem; AA = academic achievement; The variables used in the interaction terms of TS × PC and TS × PC were standardized to reduce non-essential multicollinearity. ^∗^*p* < 0.05, two-tailed. ^∗∗^*p* < 0.01, two-tailed. ^∗∗∗^*p* < 0.001, two-tailed.*

**FIGURE 2:**
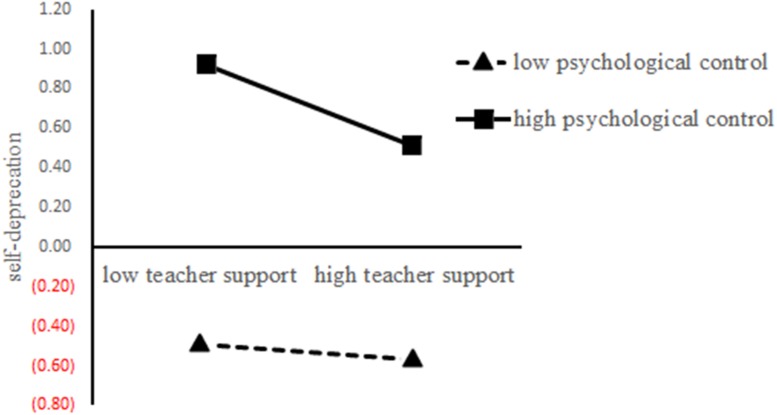
The moderating effect of psychological control on the relationship between teacher support and self-deprecation. Conditional regressions of self-deprecation on teacher support were conducted when psychological control was high (*M* + 1 *SD*) and low (*M* − 1 *SD*). Endpoints of the lines represented self-deprecation when teacher support was low or high.

Hypothesis 1 states that self-esteem mediated the positive relationship between teacher support and academic achievement. As Table 2 shows, the indirect relationship between teacher support and achievement via positive self-esteem was significant (β = 0.06, *p* < 0.01). And the indirect relationship via self-deprecation was also significant (β = 0.03, *p* < 0.01).
Hypothesis 2 states that psychological control moderates the relationship between teacher support and self-esteem. As Table 2 shows, the interaction between teacher support and psychological control did not predict positive self-esteem (β = −0.03, *p* > 0.05) but positively predicted self-deprecation (β = 0.12, *p* < 0.05). Next, using Stone and Hollenbeck’s (1989) procedure, we measured the interaction effect to self-deprecation. As is shown in Figure 2, slope test demonstrated that teacher support was negatively related to self-deprecation when psychological control is high (*r* = −0.21, *p* < 0.05) while not related to self-deprecation when psychological control is low (*r* = −0.05, *p* > 0.05).

Hypothesis 3 states the moderated mediation effect, that is, psychological control moderated the mediating effect of self-esteem on the teacher support–academic achievement relationship. Given that, psychological control did not moderate teacher support – positive self-esteem link, the hypothesis that psychological control moderated the mediating effect of positive self-esteem on the relationship was not testified. We calculated the indirect effect of teacher support on academic achievement when psychological control is high versus low. Specifically, teacher support has a positive and indirect relationship with academic achievement with high psychological control (β = 0.06, *p* < 0.05) while teacher support was not significantly related to academic achievement with low psychological control (β = 0.02, *p* > 0.05).

## Discussion

Based on SDT and EST, our study investigated both contextual factors (teacher support, psychological control) and individual factor (self-esteem) that predicted migrant children’s academic achievement, as well as the mediating role of self-esteem and the moderating role of psychological control.

As is stated in Table 2, self-esteem mediates the positive relationship between teacher support and academic achievement, which confirms prior research (Teng et al., 2017). In particular, teacher support contributes to promoting migrant children’s feelings of positive self-esteem or diminishing self-deprecation, which consequently predict their academic achievement.

Additionally, migrant children with more positive self-esteem have more confidence to cope with academic tasks and achieve their goals (Zimmerman and Cleary, 2005), whereas migrant children with self-deprecation are incompetent to deal with obstacles and accomplish their goal (Rosenberg, 1979). These findings align with SDT.

The above results indicate positive self-esteem and self-deprecation both partially mediated the impact of teacher support on migrant children’s academic achievement. Accurately, besides the indirect effect of teacher support on academic achievement through positive self-esteem and self-deprecation, teacher support is also directly connected with migrant children’s academic achievement, which suggest the vital importance of teacher support on migrant children’s academic achievement. Brown and Chu (2012) indicates that teacher’s inclusion of diversity is significantly related to academic outcome of Latino immigrant children in elementary school. Furthermore, migrant children are more sensible to teacher’s support, such as emotional support, learning support and competence support and do well academically.

Our results indicate psychological control moderates the mediating effect of self-deprecation on teacher support – academic achievement relationship, which is consistent with EST. As is shown in Figure 2, when the level of parents’ psychological control is high, the mediating effect is positive, and when psychological control is low, the mediating effect is not significant. Specifically, teacher support decreases self-deprecation and thus promote children’s academic achievement when psychological control is high. The finding suggests parental psychological control might not have such a detrimental impact, which may be related to children’s interpretation of parental “guan” as act of love and “best for children” (Chao, 1994; Helweig et al., 2014). And, high level of psychological control aligns with teacher support to decrease children’s self-deprecation, and in turn, buffer the detrimental effect of self-deprecation on their academic achievement. This finding aligns with interdependence view of Chinese culture.

However, psychological control does not moderate the mediating impact of positive self-esteem on teacher support – academic achievement relationship. Probably there might exist two possible reasons: one possible reason may be, compared with parents, teachers might play a more important role in migrant children’s positive self-esteem; the other possible reason, compared with psychological control, support might be more vital to migrant children’s positive self-esteem. Yet, Frank et al. (2010) showed that, compared to parental psychological control, parental support has a more significant impact on Iranian American adolescents’ positive self-esteem. Therefore, compared with psychological control, teacher support may be more important for migrant children’s positive self-esteem.

While this study provides a better understanding of the relations between teacher support and migrant children’s academic achievement in China, several limitations should be noted. First, with cross-sectional data, doubt about the direction of causality may be raised. Hypotheses were put forward based on SDT and EST that teacher support promotes children’s achievement by elevating their positive self-esteem and diminishing self-deprecation, and parents’ psychological control moderates the mediation link. However, it is also possible that migrant children with better achievement might receive more teacher support, possess higher self-esteem (Lee, 2012), and perceive less psychological control. Thus, longitudinal designs are needed to affirm the causal direction of the results. Second, except for academic achievement, teacher support, parents’ psychological control and self-esteem were self-report measures and thus subjected to false recall and social desirability. Although children’s self-perception showed to be highly reliable and valid (Gonzalez et al., 1996), issues with self-report measures should be considered. Future research should employ multi-informant assessment, such as children-report, parents’ report and spouse-report. Third, our sample limited to Chinese elementary children, future studies should explore to what extent the present results can be generalized to older children such as adolescents. Fourth, this study found that psychological control did not moderate the teacher support – positive self-esteem link or the mediating effect of positive self-esteem, and the finding was interpreted preliminarily based on prior empirical research findings. Thus, the relationship among teacher support, psychological control, positive self-esteem, and academic achievement requires further empirical study for verification.

Despite of the limitations above, some valuable information and important practical implications can be derived from our findings. First, the current research presents new evidence for the relationship among contextual factors, individual factors and Chinese migrant children’s academic achievement. The inclusion of teachers, parents, and children themselves broadens the literature in important ways. The findings also provide an important supplement an extension to SDT and EST. Second, the current research found that teacher support was of vital importance to migrant children’s academic achievement, and self-esteem mediated such relationship. Besides, parents’ psychological control moderated the mediating effect of self-deprecation. There is a great need for treatment programs that focus not only on teachers and parents but also on children’s self-esteem. Third, our findings indicated both cultural-universal and cultural-specific findings, and caution is required when employing western theories in the educational practice.

## Conclusion

Self-esteem mediated the positive relation between teacher support and academic achievement. Specifically, teacher support was positively related to positive self-esteem, and then, predict better academic achievement. While teacher support was negatively related to self-deprecation, and then, predicted lower academic achievement.

Parents’ psychological control moderated the relation between teacher support and self-deprecation such that teacher support negatively predicted children’s self-deprecation when the level of psychological control was high rather than low.

Parents’ psychological control moderated the mediating effect of self-deprecation on the teacher support – academic achievement relationship such that the mediating effect was positive when the level of psychological control was high rather than low.

## Data Availability Statement

The datasets generated for this study are available on request to the corresponding author.

## Ethics Statement

Before collecting questionnaires, it is necessary to acquire participants’ permission. Parental written informed consents for their children to participate were obtained.

## Author Contributions

All authors listed, have made substantial, direct and intellectual contribution to the work, and approved it for publication.

## Conflict of Interest

DZ was employed by company Shandong Dongheng Colloidal Material Co., Ltd. The remaining authors declare that the research was conducted in the absence of any commercial or financial relationships that could be construed as a potential conflict of interest.
